# Determination of Non-Digestible Parts in Dairy Cattle Feces Using U-NET and F-CRN Architectures

**DOI:** 10.3390/vetsci10010032

**Published:** 2023-01-01

**Authors:** Cevher Özden, Mutlu Bulut, Demet Çanga Boğa, Mustafa Boğa

**Affiliations:** 1 Computer Engineering, Akdeniz University, 07040 Antalya, Türkiye; 2 Department of Agricultural Engineering, Çukurova University, 01120 Adana, Türkiye; 3 Department of Chemistry and Chemical Processing, Osmaniye Korkut Ata University, 80050 Osmaniye, Türkiye; 4 Bor Vocational School, Niğde Ömer Halisdemir University, 51700 Niğde, Türkiye

**Keywords:** image processing, deep learning, livestock, images of feces, indigestible parts

## Abstract

**Simple Summary:**

This study employs Fully Convolutional Regression Networks (FCRN) and U-Shaped Convolutional Network for Image Segmentation (U-Net) architectures tailored to the dataset containing dropping images of dairy cows collected from three different private dairy farms in Nigde. The main purpose of this study is to detect the number of undigested grains in dropping images in order to give some useful feedback to raiser. It is a novel study that uses two different regression neural networks on object counting in dropping images. To our knowledge, it is the first study that counts objects in dropping images and provides information of how effectively dairy cows digest their daily rations.

**Abstract:**

Deep learning algorithms can now be used to identify, locate, and count items in an image thanks to advancements in image processing technology. The successful application of image processing technology in different fields has attracted much attention in the field of agriculture in recent years. This research was done to ascertain the number of indigestible cereal grains in animal feces using an image processing method. In this study, a regression-based way of object counting was used to predict the number of cereal grains in the feces. For this purpose, we have developed two different neural network architectures based upon Fully Convolutional Regression Networks (FCRN) and U-Net. The images used in the study were obtained from three different dairy cows enterprises operating in Nigde Province. The dataset consists of the 277 distinct dropping images of dairy cows in the farm. According to findings of the study, both models yielded quite acceptable prediction accuracy with U-Net providing slightly better prediction with a MAE value of 16.69 in the best case, compared to 23.65 MAE value of FCRN with the same batch.

## 1. Introduction

During the recent years, with the rapid advancement of image processing technology, counting and determining the location of objects in a certain area of an image has become very popular and is successfully applied in many sectors [1]. Today, locating and counting objects in an image is increasingly used in many areas such as city management [2], regulation of city traffic [3], parking lot layout [4], farm management [5], and counting fruits in a garden [6].

Object counting techniques try to most accurately determine the number of objects in an image or video segment. In contrast, object detection identifies objects of the same type in an image and pinpoints their locations [7]. The main purpose of object counting applications is to calculate the number of cars on a road or parking lot [8,9,10,11], the number of people in a crowded area [12,13,14], the number of goats and sheep in an livestock farm [15,16], and the number of apples in an cultivated area [17].

Object detection and counting is a typical computer vision task. Convolutional Neural Networks (CNNs), Support Vector Machines (SVMs), and other techniques are among the many available methods for solving the issue [18]. CNN automatically extracts the features required to solve the problem. This is one of the most important properties of convolutional networks. In traditional machine learning methods, feature extraction is not done automatically. These methods require expert knowledge. CNN architectures can be trained with large datasets, and GPU support can be provided [19]. Thanks to such advantages, artificial intelligence is also developing with the improvement of convolutional neural network methods day by day.

Object counting techniques are generally grouped under four headings: object detection and counting, counting with orbital clustering, counting with global regression, and counting based on object density estimation [20]. Object detection and counting according to the basic threshold rule is one of the most used methods. The most important advantage of this method is that it performs very fast counting, while the disadvantage is that the counting performance decreases when more than one object approaches each other [21,22,23]. In the counting process using the orbital clustering technique, crowded moving objects are counted [24,25]. This method is only applied to track objects in the direction of the desired trajectories. In addition, because rapid training and testing procedures are used in the counting process with the global regression method, it gives good results in estimations [26]. However, the number of objects to be estimated across the entire image is calculated as the integral of the density map. Unlike other methods, this method gives the density map of the object for the analysis of object distribution throughout the image [27].

A one-way regression is what the deep learning method for object counting in image processing entails. Based on training data for the number of potential output units, these networks adopt a specified categorization architecture [28]. In addition, it has a single output unit that produces a real value as close as possible to the total number of objects to be counted [29,30]. High-dimensional nonlinear regression models are a category that can be applied to both circumstances. The number of estimators that must be taken into account here is inversely related to the quantity of input pixels. Recently, with the version updates of deep learning libraries, the object counting APIs (Application Programming Interface) of Tensorflow and YOLO libraries offer practical uses [31]. Given that CNN models are capable of classifying and detecting items from a wide range of classes, counting without localizing is not difficult for these models. In reality, representations of regression-based models’ feature maps have shown that these maps contain information on object positions.

The successful application of image processing technology in different fields has attracted much attention in the field of agriculture in recent years. In this study, we focused on detecting the amount of indigestible cereal grains in animal feces using image processing methods. In animal nutrition, the digestion and absorption of feed ingredients are important for the animal’s performance and for making the most of the feed ingredients in the ration. For this purpose, fecal separators and substances that are not digested in feces should be checked from time to time under farm conditions. In the process of washing the feces with this system, the problems related to the processing of the feed and the determination of the negative conditions in the rumen conditions of the animal are among the issues to be considered. Since the use of dung washing screens (stool separators) is time consuming and requires experienced staff on the farm, such applications are difficult to implement in every farm.

In recent studies on farm animals, researchers focused on deep learning algorithms, used these algorithms with experiments, and concluded that these algorithms worked with a high percentage of success. Next-generation methods such as Mask R-CNN, Faster R-CNN, YOLO (v3 and v4), and DeepLab v3 have been used for deep learning algorithms through image processing [32]. In addition, network architectures such as ResNet, Inception, Xception, and VGG16 have shown successful results in studies on farm animals. The high success rate of deep learning algorithms improves the ability to predict ahead in different areas of livestock and provides more efficient planning. Understanding the working principles of new generation image processing techniques and choosing appropriate algorithms can save time and increase economic efficiency in livestock activities [32,33,34]. The following is a list of the study’s main contributions to the literature: (i) Counting objects using image processing technology has previously been successfully applied in car counting, people counting, and fruit counting. However, the issue of detecting indigestible substances in animal feces is not yet included in the literature. In this study, we have improved upon highly advanced CNN architectures that generate a heat map for counting non-digestible grains in feces in one go. (ii) The effect of processing stool images on the success of the heat map prediction of indigestible grains has been demonstrated by detailed experiments. (iii) In farm conditions, fecal separators and materials that are not digested in feces should be checked periodically. The use of dung washing screens (stool separators) is a time-consuming and difficult task on the farm. With this method, we have achieved the number of indigestible grains in feces with a 16.69 MAE rate. The findings we obtained in this paper indicate that the object counting method has a significant potential in solving daily tasks of dairy cow farms.

## 2. Materials and Methods

### 2.1. Material

The data used in this study was collected from the 3 different private farms with an average of 100 milking cows located in Nigde province. Dairy cattle were a fed daily total mixed ration (full feeding) with 45% roughage and 55% concentrate feed. Roughage consists of different proportions of alfalfa, wheat straw, and beet pulp; concentrated feed consists of different ratios of milk feed (19% HP, 2700 mcal energy), barley paste, and soybean meal. The dataset consists of the 277 distinct dropping images of dairy cows. The images were taken with a cellphone camera under different lighting and weather conditions. All images were resized to 256 × 256 pixels. Due to the relatively low number of images, data augmentation and upsampling methods were employed in the network architectures. Some of the original images were too large with high pixel intensity. Those images were cropped into smaller chunks of images to oversample the dataset. In addition, horizontal and vertical flip were applied for data augmentation. A total of 80% of the images were used during the training phase, and the rest were used for testing purposes. 

Supervised learning methods require labeled data for the learning phase. Therefore, all images had to be properly annotated. Most recent networks such as YOLO requires the bounding boxes that surround the object to be detected, which is a quite labor-intensive process, especially for small objects such as fruits or grains. Another approach used in the literature is regression-based estimates for counting objects instead of localizing and detecting the objects. In this approach, the problem is handled as an estimation task of a continuous density function whose integral over any region of the image yields the object count. We employed the second approach in the study. For this purpose, each undigested grain in each image was spotted by putting a red dot for each instance. In this way, the burden of detecting and localizing each object instance with a bounding box is avoided and a counting problem is approached as a regression task rather than classification. The data and the codes are shared in this public Github repository at https://github.com/cevher/Droppings (accessed on 14 November 2022). 

### 2.2. Method 

Convolutional Neural Networks are densely used for image classification and detection tasks and have achieved great success in the recent decade. They have been used for object counting tasks. The common way they work is to first detect the objects in an image and then count the found instances of the object, which is an effective method but requires the use bounding box annotations as labels during training. Labeling with a bounding box is quite hard to obtain especially for small-sized crowded objects such as the grains in droppings. The other methods used in the literature for this purpose were to count objects by estimating density heat maps much like a regression estimation. In this type of learning, dot-annotated density maps are used as labels. Dot annotation only gives the information about the location of the object and is much less labor intensive compared to bounding box annotation. As mentioned in the project of Lempitsky and Zisserman (2010) [35], the main idea is: given an image *I*, the goal is to obtain a density function *F* as a real function of pixels in the given image. Then, given the estimated density function of *F* and the query about the number of objects in the image *I*, the number of objects is estimated by integrating *F* over the entire *I.* If *F* is integrated over a subregion of the image, it gives the number of objects in this subregion [35]. The mechanism during training works in the following way: dot annotations are each represented by a Gaussian. A density surface *D(x)* is formed by the superposition of these Gaussians. The task is to regress this density surface from the corresponding cell image *I(x)*. This is achieved by training a CNN using the mean square error between the output heat map and the target density surface as the loss function for regression. The trained network then predicts the density heat map *D(x)* given an input cell image *I(x)* [36]. Fully Convolutional Regression Networks and its derivative U-Net are two most common network architectures proposed in literature for this purpose. Both are used for image segmentation in various fields such as biomedical image processing, cell counting, car counting, and pedestrian counting. Another approach could be to use pretrained models such as ResNET or VGG versions. However, the feces dataset has not been used to train such models. Therefore, in order to change the weights of the pretrained models, thousands of images would be required. Considering that we only have 277 images in the feces dataset, it would be impossible to make a reasonable change in the weights. For this reason, transfer learning is not considered in this paper.

In order to create labels for training images, each pixel in an image is connected to a real-valued feature vector for a specific set of training images. Each training image has a collection of 2D points added to it; the total number of points indicates how many items the researchers have labeled. Thus, the density functions consist of real-valued values over pixel grids. The output of applying a convolution with a Gaussian kernel across pixel grids to each input image results in a density map known as the ground truth density function, whose integrals over image regions would correspond to the item counts. Gaussian Kernel function is defined as following:

(1) $$G\left(x,y\right)={a*\exp}^{-(a{\left(x-x_{0}\right)}^{2}+2b\left(x-x_{0}\right)\left(y-y_{0}\right)+c\left(y-y_{0}{)}^{2}\right)}$$

(2) Gx,y=Gx,ymaxGx,y

**Figure 1:**
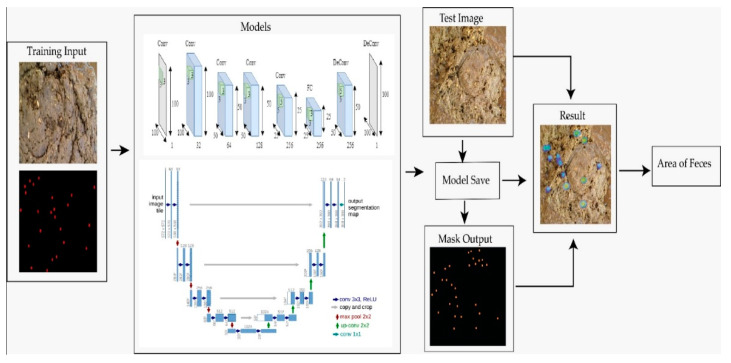
Flowchart for model implementation.

The goal is to learn the linear transformation of the feature representation that approximates the density function at each function from a set of training photos with their ground truth densities. Two distinct deep learning architectures were employed in the study to achieve this goal. One is the Fully Convolutional Regression Networks (FCRNs). Different types of this network architecture have been successfully used for solving problems such as cell counting in microscopy images. The FCRN architecture used in this study was developed upon VGG-net (Figure 1). Small kernel sizes (3 × 3 and 5 × 5 pixels) were preferred and, in this way, the number of feature maps could be increased to compensate for the loss of spatial information due to max pooling applied after every two convolutional layers in the architecture. This fine-tuning enabled the network to transfer spatial information to the higher layers of the network. The overall architecture of FCRNA consisted of two main units: Conv units and UpConv units. Each Conv unit included a convolution layer, followed by a Rectified Linear unit (ReLU) and a max-pooling; in contrast, each UpConv unit consisted of a deconvolution, ReLU, and a convolution layer. A fully connected layer was implemented after the 4th Conv unit as convolution. 

**Figure 2:**
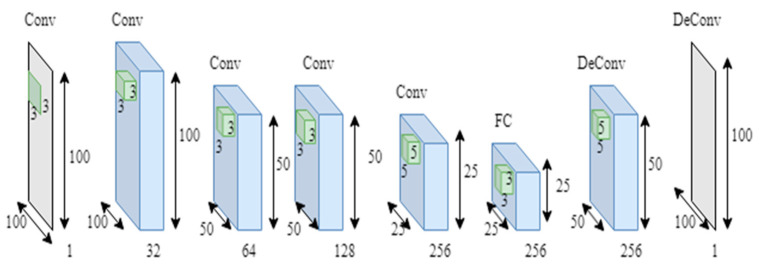
Fully Connected Regression Network Architecture (FCRN).

The second deep learning architecture is of U-Net architecture, which was built upon Fully Convolutional Networks (Figure 2). This type of network is very apt at working with a small number of training images and gives very precise segmentations. The architecture consists of two main parts: contracting and expanding paths. The contracting part of the architecture has the typical convolutional network architecture, which consists of the repeated application of two 3 × 3 convolution layers, followed by a ReLU and max pooling. This operation downsamples the given input images. The expanding part of the architecture consisted of an upsampling operation, followed by a 2 × 2 up-convolution layer. The final layer of the network is 1 × 1 convolution to map the feature vector. The network is made of 23 convolutional layers in total.

In the implementation phase, samples of 256 × 256 pixels were obtained from the center part of each dropping in the images, thus we increased the number of input images for training to some degree. In order to provide orthogonal initialization for the convolutional layer, each sample image was normalized. Stochastic Gradient Descent in PyTorch Framework was used for optimization and Mean Squared Error (MSELoss) function in PyTorch was used as loss function in both networks. The learning rate was set to 0.01, and it was decreased by 10 factors at every 10 epochs. Momentum and weight decay were taken as 0.8 and 0.005, respectively. The training was done with 200 epochs with each network. Dropout was not employed. Both U-Net and FCNR architectures employed three down sampling and three upsampling convolutional blocks with fixed 3 × 3 and 5 × 5 filter size. Each block in U-Net consisted of two convolutional layers, where FCNR started with 1 convolutional layer and the number of convolutional layers was increased by 1 for each subsequent layer in order to compensate for the loss of high-resolution information due to pooling. Both models were trained on randomly separated 80% of the image data. Upon finishing the training phase, networks were saved as pth files and they were tested on the unseen 20% of the image data. The evaluation was performed on a computer with Intel(R) Core(TM) i7-7500U CPU, 2.70 GHz. The results are given with Mean Absolute Error (MAE) and Root Mean Square Error (RMSA).

$$x_{i},y_{i\;}\to {\mathrm{actual}\;\mathrm{and}\;\mathrm{target}\;\mathrm{count}\;\mathrm{of}\;\mathrm{grains}\;\mathrm{for}\;i}^{\mathrm{th}}\mathrm{image};\;N=\mathrm{number}\;\mathrm{of}\;\mathrm{image}:$$

(3) $$\mathrm{MAE}=\frac{\sum_{i}\left|x_{i}-y_{i}\right|}{N}$$

(4) $$\mathrm{RMSE}=\sqrt{\frac{\sum_{i}{\left(x_{i}-y_{i}\right)}^{2}}{N}}$$

The most popular metrics for evaluating accuracy for continuous variables are MAE and RMSE. Both metrics are indifferent to the direction of error and can range between 0 and ∞; lower scores mean better prediction.

## 3. Results

The dataset containing photos of dairy cow droppings was subjected to the two network designs. Training sets made up 80% of the dataset, and validation sets made up 20%. The large images were randomly divided into separated sub-images to increase the amount of data. By dividing the appropriate patch’s standard deviation and removing its mean, each patch obtained in this manner was normalized. 

In RMSE, errors are squared before taking their average, which can be handy if large errors are undesirable. Both of our models were trained for 100 epochs with different batch sizes (1, 8 and 16). The obtained results are summarized for each model with respect to their MAE and RMSE scores. 

**Figure 3:**
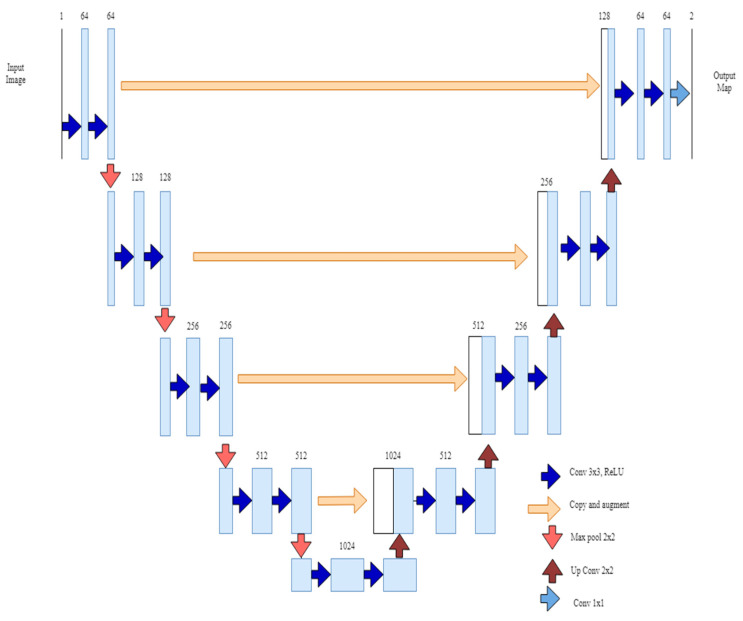
U-Net Convolutional Architecture.

From Table 1, it can be seen that U-Net architecture achieved the best MAE and RMSE values compared to FCRN in all of the different batch sizes. The lowest MAE value was achieved by U-Net with one batch size training. Sample results obtained with two architectures are given in Figure 3.

**Figure 4:**
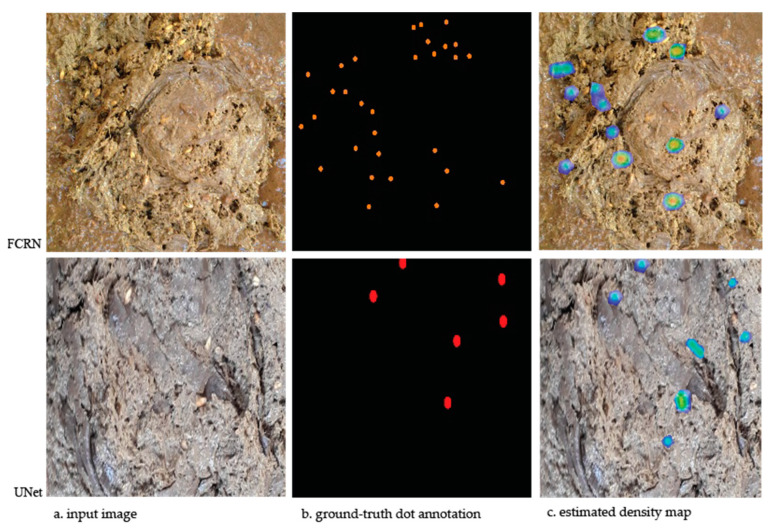
Comparison of two architecture on sample result. (**a**) Input images, (**b**) ground-truth dot annotations, (**c**) estimated density (heat) maps. FCRN: Fully Convolutional Regression Networks; U-Net: U-Shaped Convolutional Network.

Some examples of the results of both networks are given in Figure 4 Input images and ground-truth dot annotations are given as input to networks during training. Networks produce estimated density maps based on the located object centers corresponding to given input images. As can be seen, both networks closely detected density areas of undigested grains. The model summaries for FCRN and U-net are presented in Appendix A and Appendix B, respectively.

## 4. Discussion

Counting small and crowded objects of the same type in an image or video footage is a very difficult and time-consuming task. In order to overcome this problem, artificial intelligence algorithms have been used in recent years. In this study, we used Convolutional Regression Networks due to their relatively easier labelling method, which have been applied in recent years for counting objects in an image. Different CRN architectures have been used in various researches in the field of biology, remote sensing, surveillance, etc. It is thought that the method we used in our study is new in the field of animal husbandry and the need for such applications has increased recently. For this reason, we predict that the interest in this subject and the number of these studies may increase in the future. However, due to the lack of studies on the determination of indigestible products in the feces of animals, a comparison could not be made in this study. However, some studies using this architecture are briefly summarized for comparison purposes in the following paragraph. 

The results of the study are quite acceptable when compared to other studies in the literature that implement different regression network architectures on various datasets. One of them is Õnoro-Rubio and López-Sastre’s (2016) [37] study. They proposed Counting Convolution Neural Network and Hydra Convolutional Neural Network models and tested them on the TRANCOS dataset, which is a publicly available dataset of 1244 images of different traffic scenes. Their MAE results ranged between 10.99 and 25.99. A recent study was carried out by Kilic and Ozturk [38], who proposed a new architecture called Heatmap Learner Convolutional Neural Network (HLCNN) developed upon VGG-16. They conducted their tests on CARPK and PUCPR+ datasets, which are publicly available car images captured in a parking lot. They compared their results with the results of previous studies using the same datasets. They reported a state-of-the-art result as 2.12 and 2.52 MAE values; the previous studies’ MAE results ranged between 4.45 and 156.00. Pedestrian and car datasets have been heavily researched in most of the previous studies. Xie et al. (2018) [36] used CRN to count microscopic cells and they revealed that CRNs made quite good predictions without fine-tuning the count of cells in real microscope images. It has also been stated that it is possible to obtain better results by fine-tuning the CRN performance to the images. Haider et al. (2021) [39] used the Fully Convolutional Regression Network architecture for human detection from aerial thermal camera images. Two well-constructed models called FCN and U-net were chosen to compare the detection performance. It has been found that the method used in that study can detect people in crowded images with high precision, and that it provides better results compared to new techniques that detect people using thermal imaging. The precision estimate of the FCRN architecture proposed in the study was found to be 99%. Kılıç and Özturk (2021) [38] tried to estimate an exact number of cars in aerial images using convolutional neural network architecture. CNN architecture was developed by adding three convolutional adaptation layers to determine the heat maps of the cars to be counted. In the experiments, it has been shown that the developed model gives very successful results in determining the points where the cars are located. In addition, it was emphasized that more successful results were obtained thanks to the Heatmap CNN compared to the existing object counting techniques.

The current paper is the first study implemented on dropping images. Despite this fact, the results we achieved in this study are quite close to the results of the previous studies implemented on other image datasets. Sample results obtained with two architectures are given in Figure 3. When the literature is examined, it can be seen that a number of studies examining the counting of objects are classified into two main groups: counting by detection and regression. Object detection system inculcates CNN models to reveal the properties of sample object suggestions and then estimate the class probabilities and regression bounding boxes of those suggestions. These methods are defined as two-stage object detection, and the best known methods belong to the R-CNN family [40,41]. While two-stage methods are somewhat successful in detecting objects, single-shot methods such as YOLO and SSD provide a faster experience [42,43].

Object detection is considered a useful application of deep neural networks because it significantly achieves visual-based detection sensitivity. High-dimensional images are processed with the help of convolutional neural networks so that they can have strong substantiality and endurance to noise and deficit [44]. It has been determined that deep convolutional neural networks are used in many studies for agricultural purposes. In the studies of Kamileris and Prenafeta-Boldú (2018) [45], 40 papers using the words deep learning and agriculture were identified and these articles were evaluated with bibliographic analysis. In the study, it was reported that deep learning methods achieved higher detection sensitivity compared to previous image processing methods. Mirhaji et al. (2021) [46] carried out a study on oranges using image processing technology under various lighting conditions. The YOLOv4 network, one of the image processing techniques, was used in the study and almost 90% success was achieved. In a similar study, Koirala et al. (2019) [47] used RCNN from the YOLO series. This method has achieved an 80% success in detecting fruits on trees.

## 5. Conclusions

In this paper, two different architectures were used for the detection of indigestible substances in the feces of dairy cows. A heatmap-generated CNN architecture was used to count the indigestible fractions in the feces of dairy cows in a single shot. After computer processing the feces images, the effect of indigestible fractions on the success of the heatmap estimation is shown. Under farm conditions, indigestible materials should be checked during the day with feces sieves. It is quite difficult to collect fecal data in livestock enterprises. In addition, the difficulties in the processing and evaluation of the obtained data and the inhomogeneity of the data limits the number of such studies and these conditions are the fundamental limitations of the study. However, it is important to visually determine the indigestible parts of feces in animal feeding. In order to determine this, stool sieves are widely used in Turkey. However, it takes an average of 5–7 min to sift the feces of each animal, which is challenging work. In this case, it would be possible to examine a few feces in the farm in general, but not the whole farm. Examination of a few feces will not cover the entire farm and will prevent a clear idea of the entire herd from forming. For this reason, by using FCRN architecture in our study, it will be possible to evaluate the feces in the whole farm in a short time to get faster results and to prevent the problem of determining the indigestible parts of the feces. Providing fast and accurate results in a short time with an artificial intelligence method will lead to more profitable livestock and more performance increases in the farm, which are the main contributions of the current study of the dairy industry. The method will enable the detection of the negativities in the processing and preparation of feeds for animals, and any problem can be eliminated on short notice. In addition, with this method, the negative effects of the animal’s digestive system can be determined and it would be possible to take nutritional measures with the ration. This timely intervention will increase the profitability of the farm by providing timely prevention of health problems and could increase performance. These results show that the object counting method has significant potential in solving the daily tasks of dairy farms more effectively.

## Figures and Tables

**Table 1:** FCRN and U-Net Results.

Network	Batch Size	MAE	RMSE
FCRN	1	23.65	36.69
	8	37.78	45.76
	16	158.71	201.46
U-Net	1	16.69	22.48
	8	24.42	34.64
	16	144.28	198.34

MAE: Mean Absolute Error; RMSE: Root mean squared error. The lower scores mean better prediction. FCRN: Fully Convolutional Regression Networks; U-Net: U-Shaped Convolutional Network.

## Data Availability

The data and the codes are shared in this public Github repository (https://github.com/cevher/Droppings). Accessed on 14 November 2022.

## Appendix A. Model Summary for FCRN

**Layer (Type)**	**Output Shape**	**Param #**
Conv2d-1	[−1, 32, 256, 256]	864
BatchNorm2d-2	[−1, 32, 256, 256]	64
ReLU-3	[−1, 32, 256, 256]	0
MaxPool2d-4	[−1, 32, 128, 128]	0
Conv2d-5	[−1, 64, 128, 128]	18,432
BatchNorm2d-6	[−1, 64, 128, 128]	128
ReLU-7	[−1, 64, 128, 128]	0
MaxPool2d-8	[−1, 64, 64, 64]	0
Conv2d-9	[−1, 128, 64, 64]	73,728
BatchNorm2d-10	[−1, 128, 64, 64]	256
ReLU-11	[−1, 128, 64, 64]	0
MaxPool2d-12	[−1, 128, 32, 32]	0
Conv2d-13	[−1, 512, 32, 32]	589,824
BatchNorm2d-14	[−1, 512, 32, 32]	1024
ReLU-15	[−1, 512, 32, 32]	0
Upsample-16	[−1, 512, 64, 64]	0
Conv2d-17	[−1, 128, 64, 64]	589,824
BatchNorm2d-18	[−1, 128, 64, 64]	256
ReLU-19	[−1, 128, 64, 64]	0
Upsample-20	[−1, 128, 128, 128]	0
Conv2d-21	[−1, 64, 128, 128]	73,728
BatchNorm2d-22	[−1, 64, 128, 128]	128
ReLU-23	[−1, 64, 128, 128]	0
Upsample-24	[−1, 64, 256, 256]	0
Conv2d-25	[−1, 1, 256, 256]	576
BatchNorm2d-26	[−1, 1, 256, 256]	2
ReLU-27	[−1, 1, 256, 256]	0
Total params: 1,348,834 Trainable params: 134,883		

## Appendix B. Model Summary for U-Net

**Layer (Type)**	**Output Shape**	**Param #**
Conv2d-1	[−1, 64, 256, 256]	1728
BatchNorm2d-2	[−1, 64, 256, 256]	128
ReLU-3	[−1, 64, 256, 256]	0
Conv2d-4	[−1, 64, 256, 256]	36,864
BatchNorm2d-5	[−1, 64, 256, 256]	128
ReLU-6	[−1, 64, 256, 256]	0
Conv2d-7	[−1, 64, 128, 128]	36,864
BatchNorm2d-8	[−1, 64, 128, 128]	128
ReLU-9	[−1, 64, 128, 128]	0
Conv2d-10	[−1, 64, 128, 128]	36,864
BatchNorm2d-11	[−1, 64, 128, 128]	128
ReLU-12	[−1, 64, 128, 128]	0
Conv2d-13	[−1, 64, 64, 64]	36,864
BatchNorm2d-14	[−1, 64, 64, 64]	128
ReLU-15	[−1, 64, 64, 64]	0
Conv2d-16	[−1, 64, 64, 64]	36,864
BatchNorm2d-17	[−1, 64, 64, 64]	128
ReLU-18	[−1, 64, 64, 64]	0
Conv2d-19	[−1, 64, 32, 32]	36,864
BatchNorm2d-20	[−1, 64, 32, 32]	128
ReLU-21	[−1, 64, 32, 32]	0
Conv2d-22	[−1, 64, 32, 32]	36,864
BatchNorm2d-23	[−1, 64, 32, 32]	128
ReLU-24	[−1, 64, 32, 32]	0
Upsample-25	[−1, 64, 64, 64]	0
ConvCat-26	[−1, 128, 64, 64]	0
Conv2d-27	[−1, 64, 64, 64]	73,728
BatchNorm2d-28	[−1, 64, 64, 64]	128
ReLU-29	[−1, 64, 64, 64]	0
Conv2d-30	[−1, 64, 64, 64]	36,864
BatchNorm2d-31	[−1, 64, 64, 64]	128
ReLU-32	[−1, 64, 64, 64]	0
Upsample-33	[−1, 64, 128, 128]	0
ConvCat-34	[−1, 128, 128, 128]	0
Conv2d-35	[−1, 64, 128, 128]	73,728
BatchNorm2d-36	[−1, 64, 128, 128]	128
ReLU-37	[−1, 64, 128, 128]	0
Conv2d-38	[−1, 64, 128, 128]	36,864
BatchNorm2d-39	[−1, 64, 128, 128]	128
ReLU-40	[−1, 64, 128, 128]	0
Upsample-41	[−1, 64, 256, 256]	0
ConvCat-42	[−1, 128, 256, 256]	0
Conv2d-43	[−1, 64, 256, 256]	73,728
BatchNorm2d-44	[−1, 64, 256, 256]	128
ReLU-45	[−1, 64, 256, 256]	0
Conv2d-46	[−1, 64, 256, 256]	36,864
BatchNorm2d-47	[−1, 64, 256, 256]	128
ReLU-48	[−1, 64, 256, 256]	0
Conv2d-49	[−1, 1, 256, 256]	64
Total params: 593,408 Trainable params: 593,408		
